# *N*-(2,6-Dimethyl­phen­yl)-2-methyl­benzamide

**DOI:** 10.1107/S160053680802309X

**Published:** 2008-07-26

**Authors:** B. Thimme Gowda, Sabine Foro, B. P. Sowmya, Hartmut Fuess

**Affiliations:** a Department of Chemistry, Mangalore University, Mangalagangotri 574 199, Mangalore, India; b Institute of Materials Science, Darmstadt University of Technology, Petersenstrasse 23, D-64287 Darmstadt, Germany

## Abstract

In the title mol­ecule, C_16_H_17_NO, the N—H and C=O groups are in the anti­periplanar conformation that has been observed in related compounds. Furthermore, the conformation of the C=O group with respect to the methyl substituent in the 2-methyl­phenyl ring is *syn*, as has also been observed in related structures. The amide group makes dihedral angles of 50.3 (3) and 64.6 (3)° with the 2-methyl­phenyl and 2,6-dimethyl­phenyl rings, respectively, while the angle between the planes of the two rings is 14.26 (7)°. The mol­ecules are packed into chains *via* N—H⋯O hydrogen bonds. An intramolecular C—H⋯O hydrogen bond is also observed.

## Related literature

For related literature, see: Gowda *et al.* (2003 ▶); Gowda, Foro *et al.* (2008 ▶); Gowda, Tokarčík *et al.* (2008 ▶).
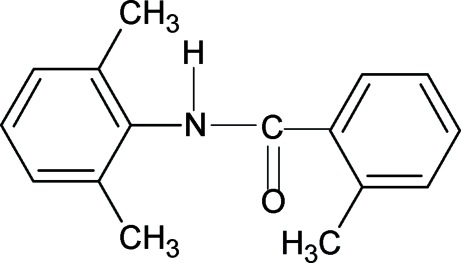


## Experimental

### 

#### Crystal data

C_16_H_17_NO
*M* *_r_* = 239.31
Orthorhombic, 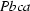

*a* = 11.687 (1) Å
*b* = 10.0187 (8) Å
*c* = 22.108 (2) Å
*V* = 2588.6 (4) Å^3^
*Z* = 8
Mo *K*α radiation
μ = 0.08 mm^−1^
*T* = 100 (2) K
0.36 × 0.24 × 0.04 mm

#### Data collection

Oxford Xcalibur diffractometer with Sapphire CCD detector
Absorption correction: multi-scan (*CrysAlis RED*; Oxford Diffraction, 2007 ▶) *T*_min_ = 0.971, *T*_max_ = 0.999
10773 measured reflections
2624 independent reflections
1864 reflections with *I* > 2σ(*I*)
*R*_int_ = 0.024

#### Refinement

*R*[*F* ^2^ > 2σ(*F* ^2^)] = 0.036
*wR*(*F* ^2^) = 0.127
*S* = 1.00
2624 reflections
169 parameters
H atoms treated by a mixture of independent and constrained refinement
Δρ_max_ = 0.27 e Å^−3^
Δρ_min_ = −0.21 e Å^−3^

### 

Data collection: *CrysAlis CCD* (Oxford Diffraction, 2004 ▶); cell refinement: *CrysAlis RED* (Oxford Diffraction, 2007 ▶); data reduction: *CrysAlis RED*; program(s) used to solve structure: *SHELXS97* (Sheldrick, 2008 ▶); program(s) used to refine structure: *SHELXL97* (Sheldrick, 2008 ▶) and *JANA2000* (Petříček *et al.*, 2000 ▶); molecular graphics: *PLATON* (Spek, 2003 ▶); software used to prepare material for publication: *SHELXS97*.

## Supplementary Material

Crystal structure: contains datablocks I, global. DOI: 10.1107/S160053680802309X/fb2099sup1.cif

Structure factors: contains datablocks I. DOI: 10.1107/S160053680802309X/fb2099Isup2.hkl

Additional supplementary materials:  crystallographic information; 3D view; checkCIF report

## Figures and Tables

**Table 1:** Hydrogen-bond geometry (Å, °)

*D*—H⋯*A*	*D*—H	H⋯*A*	*D*⋯*A*	*D*—H⋯*A*
N1—H1N⋯O1^i^	0.917 (17)	2.012 (17)	2.9248 (15)	173.7 (14)
C15—H15*A*⋯O1	0.98	2.53	3.1170 (17)	118

Symmetry code: (i) 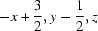.

**Fig. 1.**
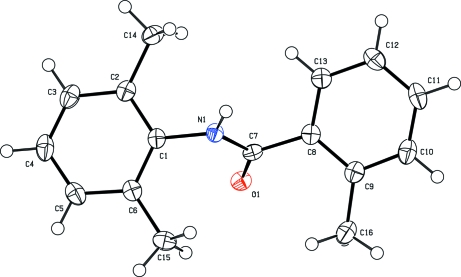
Molecular structure of the title compound, showing the atom labelling scheme. The displacement ellipsoids are drawn at the 50% probability level. The H atoms are represented as small spheres of arbitrary radii.

**Fig. 2.**
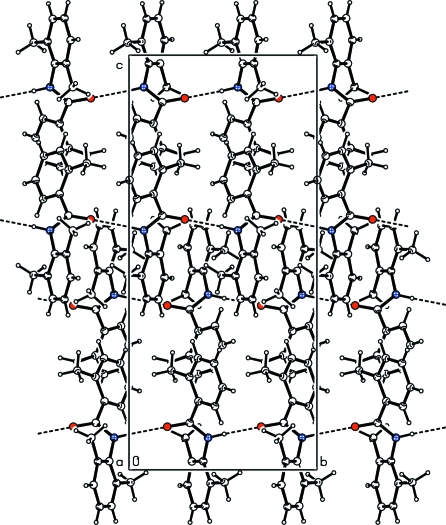
Molecular packing of the title compound with hydrogen bonding shown as dashed lines.

## supplementary crystallographic information

### Comment

In the present work, the structure of 2-methyl-*N*-(2,6-dimethylphenyl)-benzamide (N26DMP2MBA) has been determined in order to explore the effect of the substituents on the structures of benzanilides (Gowda *et al.*, 2003; Gowda, Foro *et al.*, 2008; Gowda, Tokarčík *et al.*, 2008). In the structure of the title compound (N26DMP2MBA) (Fig. 1), the N—H and C═O groups are in antiperiplanar conformation. This conformation is similar to the conformations in the already determined structures, *e.g.* in 2-methyl-*N*-(phenyl)-benzamide (NP2MBA) (Gowda, Foro *et al.*, 2008); in 2-methyl-*N*-(2-methylphenyl)-benzamide (N2MP2MBA) and in *N*-(2,6-dimethylphenyl)-benzamide (N26DMPBA) (Gowda, Tokarčík *et al.*, 2008). Further, in the title compound N26DMP2MBA, the conformation of the C═O group to the methyl substituent in the 2-methylphenyl ring is *syn*. This conformation is similar to those observed in NP2MBA and N2MP2MBA. The bond distances and angles in N26DMP2MBA are similar to those in NP2MBA, N2MP2MBA, N26DMPBA and other benzanilides (Gowda *et al.*, 2003; Gowda, Foro *et al.*, 2008; Gowda, Tokarčík *et al.*, 2008). The amide group makes the dihedral angles equal to 50.3 (3)° and 64.6 (3)° with the 2-methylphenyl and 2,6-dimethylphenyl rings, respectively, while the angle between the planes of both rings is 14.26 (7)°. In the crystal structure, the molecules are linked into chains *via* intermolecular N—H···O hydrogen bonds (Table 1). These chains are parallel to the *a* axis (Fig. 2).

### Experimental

The title compound was prepared according to the method described by Gowda *et al.* (2003). The purity of the compound was checked by determining its melting point (136°C). The title compound was also characterized by recording its infrared and NMR spectra. Plate-like colourless layered crystals with edges in the range from 0.2 to 1.0 mm were obtained by slow evaporation at room temperature from an ethanol solution (0.5 g of the title compound in about 40 ml of ethanol).

### Refinement

All the hydrogen atoms could have been discerned in the difference Fourier map, nevertheless, all the H atoms attached to the carbon atoms were constrained in a riding motion approximation with C_aryl_—H = 0.95, C_methyl_—H = 0.98 Å, while *U*_iso_H = 1.2*U*_eq_C. The positional parameters of H_N_ were refined freely. *U*_iso_H_N_ = 1.2*U*_eq_N. Five not matching reflections (2 0 0; 2 1 1; 1 0 2; 1 1 2; 1 1 3) were omitted from the refinement since their |F_o_-F_c_)|/σ(F_o_)>100 (Petříček *et al.*, 2000).

### Crystal data

**Table:**

C_16_H_17_NO	*D*_x_ = 1.228 Mg m^−^^3^
*M**_r_* = 239.31	Melting point: 409 K
Orthorhombic, *P**b**c**a*	Mo *K*α radiation λ = 0.71073 Å
Hall symbol: -P 2ac 2ab	Cell parameters from 4403 reflections
*a* = 11.687 (1) Å	θ = 2.2–28.0º
*b* = 10.0187 (8) Å	µ = 0.08 mm^−^^1^
*c* = 22.108 (2) Å	*T* = 100 (2) K
*V* = 2588.6 (4) Å^3^	Plate, colourless
*Z* = 8	0.36 × 0.24 × 0.04 mm
*F*_000_ = 1024	

### Data collection

**Table:**

Oxford Xcalibur diffractometer with Sapphire CCD detector	2624 independent reflections
Radiation source: fine-focus sealed tube	1864 reflections with *I* > 2σ(*I*)
Monochromator: graphite	*R*_int_ = 0.024
*T* = 100(2) K	θ_max_ = 26.4º
Rotation method data acquisition using ω and φ scans	θ_min_ = 2.8º
Absorption correction: multi-scan(CrysAlis RED; Oxford Diffraction, 2007)	*h* = −14→14
*T*_min_ = 0.971, *T*_max_ = 0.999	*k* = −11→12
10773 measured reflections	*l* = −27→27

### Refinement

**Table:**

Refinement on *F*^2^	Secondary atom site location: difference Fourier map
Least-squares matrix: full	Hydrogen site location: difference Fourier map
*R*[*F*^2^ > 2σ(*F*^2^)] = 0.037	H atoms treated by a mixture of independent and constrained refinement
*wR*(*F*^2^) = 0.127	*w* = 1/[σ^2^(*F*_o_^2^) + (0.0871*P*)^2^ + 0.016*P*] where *P* = (*F*_o_^2^ + 2*F*_c_^2^)/3
*S* = 1.00	(Δ/σ)_max_ < 0.001
2624 reflections	Δρ_max_ = 0.27 e Å^−^^3^
169 parameters	Δρ_min_ = −0.21 e Å^−^^3^
62 constraints	Extinction correction: none
Primary atom site location: structure-invariant direct methods	

### Special details

**Table:**

Experimental. CrysAlis RED, Oxford Diffraction Ltd., 2007 Empirical absorption correction using spherical harmonics, implemented in SCALE3 ABSPACK scaling algorithm.
Geometry. All e.s.d.'s (except the e.s.d. in the dihedral angle between two l.s. planes) are estimated using the full covariance matrix. The cell e.s.d.'s are taken into account individually in the estimation of e.s.d.'s in distances, angles and torsion angles; correlations between e.s.d.'s in cell parameters are only used when they are defined by crystal symmetry. An approximate (isotropic) treatment of cell e.s.d.'s is used for estimating e.s.d.'s involving l.s. planes.
Refinement. Refinement of *F*^2^ against ALL reflections. The weighted *R*-factor *wR* and goodness of fit *S* are based on *F*^2^, conventional *R*-factors *R* are based on *F*, with *F* set to zero for negative *F*^2^. The threshold expression of *F*^2^ > σ(*F*^2^) is used only for calculating *R*-factors(gt) *etc*. and is not relevant to the choice of reflections for refinement. *R*-factors based on *F*^2^ are statistically about twice as large as those based on *F*, and *R*- factors based on ALL data will be even larger.

### Fractional atomic coordinates and isotropic or equivalent isotropic displacement parameters (Å^2^)

**Table:**

	*x*	*y*	*z*	*U*_iso_*/*U*_eq_	
C1	0.66409 (12)	0.10525 (13)	0.52066 (6)	0.0192 (3)	
C2	0.71926 (13)	0.06605 (13)	0.46747 (6)	0.0220 (3)	
C3	0.66617 (14)	0.09420 (15)	0.41272 (7)	0.0286 (4)	
H3	0.7031	0.0707	0.3760	0.034*	
C4	0.56017 (15)	0.15594 (14)	0.41088 (7)	0.0311 (4)	
H4	0.5253	0.1753	0.3731	0.037*	
C5	0.50550 (13)	0.18914 (14)	0.46372 (7)	0.0275 (4)	
H5	0.4319	0.2291	0.4620	0.033*	
C6	0.55589 (12)	0.16532 (13)	0.52008 (6)	0.0214 (3)	
C7	0.76049 (11)	0.17701 (13)	0.61334 (6)	0.0188 (3)	
C8	0.83330 (12)	0.13044 (13)	0.66513 (6)	0.0204 (3)	
C9	0.81566 (13)	0.17869 (13)	0.72420 (7)	0.0238 (3)	
C10	0.88936 (13)	0.13316 (14)	0.76907 (7)	0.0302 (4)	
H10	0.8775	0.1622	0.8095	0.036*	
C11	0.97939 (14)	0.04706 (16)	0.75717 (7)	0.0318 (4)	
H11	1.0285	0.0188	0.7889	0.038*	
C12	0.99719 (13)	0.00269 (15)	0.69892 (8)	0.0298 (4)	
H12	1.0596	−0.0549	0.6901	0.036*	
C13	0.92333 (13)	0.04273 (14)	0.65328 (7)	0.0244 (3)	
H13	0.9342	0.0100	0.6134	0.029*	
C14	0.83131 (13)	−0.00795 (15)	0.46972 (7)	0.0277 (4)	
H14A	0.8217	−0.0904	0.4930	0.033*	
H14B	0.8893	0.0482	0.4891	0.033*	
H14C	0.8558	−0.0298	0.4285	0.033*	
C15	0.49430 (12)	0.20052 (15)	0.57709 (7)	0.0270 (4)	
H15A	0.5274	0.2820	0.5943	0.032*	
H15B	0.5019	0.1273	0.6062	0.032*	
H15C	0.4131	0.2154	0.5682	0.032*	
C16	0.72240 (14)	0.27522 (16)	0.73949 (7)	0.0319 (4)	
H16A	0.6542	0.2545	0.7155	0.038*	
H16B	0.7479	0.3662	0.7304	0.038*	
H16C	0.7040	0.2682	0.7826	0.038*	
O1	0.74295 (8)	0.29638 (10)	0.60380 (4)	0.0221 (3)	
N1	0.71980 (10)	0.07909 (12)	0.57731 (5)	0.0198 (3)	
H1N	0.7370 (13)	−0.0079 (17)	0.5864 (7)	0.024*	

### Atomic displacement parameters (Å^2^)

**Table:**

	*U*^11^	*U*^22^	*U*^33^	*U*^12^	*U*^13^	*U*^23^
C1	0.0241 (7)	0.0121 (7)	0.0214 (7)	−0.0040 (6)	−0.0027 (6)	0.0008 (5)
C2	0.0294 (8)	0.0141 (7)	0.0226 (8)	−0.0037 (6)	−0.0004 (6)	0.0003 (5)
C3	0.0437 (10)	0.0198 (8)	0.0223 (7)	−0.0024 (7)	−0.0011 (7)	−0.0009 (6)
C4	0.0469 (10)	0.0206 (8)	0.0259 (8)	−0.0016 (8)	−0.0131 (7)	0.0003 (6)
C5	0.0293 (8)	0.0167 (7)	0.0364 (9)	0.0004 (7)	−0.0101 (7)	−0.0005 (6)
C6	0.0247 (7)	0.0128 (7)	0.0266 (8)	−0.0047 (6)	−0.0035 (6)	0.0012 (6)
C7	0.0199 (7)	0.0159 (8)	0.0208 (7)	−0.0020 (6)	0.0058 (6)	0.0001 (6)
C8	0.0243 (7)	0.0141 (7)	0.0228 (8)	−0.0052 (6)	−0.0003 (6)	0.0029 (5)
C9	0.0284 (7)	0.0180 (7)	0.0251 (8)	−0.0050 (6)	−0.0010 (6)	−0.0009 (6)
C10	0.0403 (9)	0.0270 (8)	0.0235 (8)	−0.0038 (7)	−0.0063 (7)	−0.0011 (6)
C11	0.0337 (8)	0.0290 (8)	0.0326 (9)	−0.0019 (7)	−0.0136 (7)	0.0053 (7)
C12	0.0266 (8)	0.0256 (8)	0.0372 (10)	0.0017 (7)	−0.0050 (7)	0.0027 (7)
C13	0.0280 (8)	0.0184 (7)	0.0267 (8)	−0.0009 (7)	−0.0011 (6)	0.0009 (6)
C14	0.0339 (8)	0.0245 (8)	0.0246 (8)	0.0035 (7)	0.0050 (7)	−0.0006 (6)
C15	0.0242 (7)	0.0223 (8)	0.0346 (9)	0.0002 (7)	0.0030 (7)	0.0012 (6)
C16	0.0402 (9)	0.0327 (9)	0.0227 (8)	−0.0003 (8)	−0.0003 (7)	−0.0037 (7)
O1	0.0275 (6)	0.0139 (5)	0.0250 (6)	−0.0004 (4)	0.0020 (4)	0.0014 (4)
N1	0.0260 (6)	0.0142 (6)	0.0193 (6)	0.0019 (5)	−0.0025 (5)	0.0018 (5)

### Geometric parameters (Å, °)

**Table:**

C1—C2	1.397 (2)	C9—C16	1.496 (2)
C1—C6	1.400 (2)	C10—C11	1.386 (2)
C1—N1	1.4357 (17)	C10—H10	0.9500
C2—C3	1.389 (2)	C11—C12	1.378 (2)
C2—C14	1.506 (2)	C11—H11	0.9500
C3—C4	1.385 (2)	C12—C13	1.387 (2)
C3—H3	0.9500	C12—H12	0.9500
C4—C5	1.372 (2)	C13—H13	0.9500
C4—H4	0.9500	C14—H14A	0.9800
C5—C6	1.399 (2)	C14—H14B	0.9800
C5—H5	0.9500	C14—H14C	0.9800
C6—C15	1.494 (2)	C15—H15A	0.9800
C7—O1	1.2316 (17)	C15—H15B	0.9800
C7—N1	1.3502 (18)	C15—H15C	0.9800
C7—C8	1.5009 (19)	C16—H16A	0.9800
C8—C13	1.396 (2)	C16—H16B	0.9800
C8—C9	1.408 (2)	C16—H16C	0.9800
C9—C10	1.391 (2)	N1—H1N	0.917 (17)
			
C2—C1—C6	121.98 (13)	C12—C11—C10	119.52 (14)
C2—C1—N1	118.27 (12)	C12—C11—H11	120.2
C6—C1—N1	119.73 (12)	C10—C11—H11	120.2
C3—C2—C1	118.04 (13)	C11—C12—C13	119.50 (15)
C3—C2—C14	121.15 (13)	C11—C12—H12	120.2
C1—C2—C14	120.78 (12)	C13—C12—H12	120.2
C4—C3—C2	121.05 (14)	C12—C13—C8	120.98 (14)
C4—C3—H3	119.5	C12—C13—H13	119.5
C2—C3—H3	119.5	C8—C13—H13	119.5
C5—C4—C3	119.97 (14)	C2—C14—H14A	109.5
C5—C4—H4	120.0	C2—C14—H14B	109.5
C3—C4—H4	120.0	H14A—C14—H14B	109.5
C4—C5—C6	121.39 (14)	C2—C14—H14C	109.5
C4—C5—H5	119.3	H14A—C14—H14C	109.5
C6—C5—H5	119.3	H14B—C14—H14C	109.5
C5—C6—C1	117.49 (13)	C6—C15—H15A	109.5
C5—C6—C15	120.57 (13)	C6—C15—H15B	109.5
C1—C6—C15	121.93 (12)	H15A—C15—H15B	109.5
O1—C7—N1	123.09 (13)	C6—C15—H15C	109.5
O1—C7—C8	121.79 (12)	H15A—C15—H15C	109.5
N1—C7—C8	115.08 (12)	H15B—C15—H15C	109.5
C13—C8—C9	120.04 (13)	C9—C16—H16A	109.5
C13—C8—C7	118.69 (12)	C9—C16—H16B	109.5
C9—C8—C7	121.19 (12)	H16A—C16—H16B	109.5
C10—C9—C8	117.28 (14)	C9—C16—H16C	109.5
C10—C9—C16	120.15 (13)	H16A—C16—H16C	109.5
C8—C9—C16	122.57 (13)	H16B—C16—H16C	109.5
C11—C10—C9	122.62 (14)	C7—N1—C1	122.80 (12)
C11—C10—H10	118.7	C7—N1—H1N	118.9 (10)
C9—C10—H10	118.7	C1—N1—H1N	117.7 (10)
			
C6—C1—C2—C3	3.1 (2)	N1—C7—C8—C9	−132.73 (14)
N1—C1—C2—C3	−178.62 (12)	C13—C8—C9—C10	−1.3 (2)
C6—C1—C2—C14	−175.18 (12)	C7—C8—C9—C10	−178.09 (12)
N1—C1—C2—C14	3.1 (2)	C13—C8—C9—C16	178.58 (14)
C1—C2—C3—C4	−1.8 (2)	C7—C8—C9—C16	1.8 (2)
C14—C2—C3—C4	176.51 (13)	C8—C9—C10—C11	2.0 (2)
C2—C3—C4—C5	−0.6 (2)	C16—C9—C10—C11	−177.92 (14)
C3—C4—C5—C6	1.8 (2)	C9—C10—C11—C12	−0.7 (2)
C4—C5—C6—C1	−0.5 (2)	C10—C11—C12—C13	−1.3 (2)
C4—C5—C6—C15	−179.17 (13)	C11—C12—C13—C8	1.9 (2)
C2—C1—C6—C5	−2.0 (2)	C9—C8—C13—C12	−0.6 (2)
N1—C1—C6—C5	179.75 (12)	C7—C8—C13—C12	176.27 (13)
C2—C1—C6—C15	176.66 (13)	O1—C7—N1—C1	7.7 (2)
N1—C1—C6—C15	−1.6 (2)	C8—C7—N1—C1	−169.99 (11)
O1—C7—C8—C13	−127.30 (14)	C2—C1—N1—C7	112.35 (15)
N1—C7—C8—C13	50.44 (17)	C6—C1—N1—C7	−69.34 (18)
O1—C7—C8—C9	49.54 (19)		

### Hydrogen-bond geometry (Å, °)

**Table:**

*D*—H···*A*	*D*—H	H···*A*	*D*···*A*	*D*—H···*A*
N1—H1N···O1^i^	0.917 (17)	2.012 (17)	2.9248 (15)	173.7 (14)
C15—H15A···O1	0.98	2.53	3.1170 (17)	118

Symmetry codes: (i) −*x*+3/2, *y*−1/2, *z*.
